# Supplementary material for: Regulation of photosynthetic electron flow on dark to light transition by ferredoxin:NADP(H) oxidoreductase interactions
Source: eLife. 2021 Mar 9;10:e56088. doi: 10.7554/eLife.56088 (PMC7984839; doi:10.7554/eLife.56088)
Supplement: Supplementary file 1. — Taken from Hanke et al., 2005, Okutani et al., 2005, Lintala et al., 2009, Lintala et al., 2007, and Twachtmann et al., 2012. Kinetic parameters are for the reverse direction to photosynthesis (NADPH-dependent reduction of Fd). [file elife-56088-supp1.docx]

| Supplementary File 1. Summary of published information about Arabidopsis and maize FNR iso-proteins. Taken from Hanke *et al*., (2015); Okutani *et al*., (2005), Lintalla *et al*., (2007) Lintalla et al (2009) and Twachtmann *et al*., (2012). Kinetic parameters are for the reverse direction to photosynthesis (NADPH dependent reduction of Fd). | | | | | | | | | | | | | |
| --- | --- | --- | --- | --- | --- | --- | --- | --- | --- | --- | --- | --- | --- |
| *Arabidopsis* | | | | | | | | | | | | | |
|  | *enzymatic* | | |  | *Localisation in Arabidopsis* | | | | |  | *genetic analysis* | *citation* |  |
| FNR isoforms | *K*_M_ Fd µM | *k*_cat_  s^-1^ | pI | | location, on cell rupture | Cyt_b_*f* co-purification | Arabidopsis tether | | | | Impact of mutation / knock down |  |  |
| AtFNR1 | 3.5, 2.5 | - | 5.5 | | membrane bound and soluble | not determined | Tic62 / TROL | | | | remaining AtFNR2 becomes soluble | 1, 3, 4 |  |
| AtFNR2 | 4.3, 3.5 | - | 6.2 | | membrane bound and soluble | not determined | Tic62 / TROL | | | | remaining AtFNR1 is partly membrane bound | 1, 4 |  |
| *Zea mays* | | | | |  | |  | |  | | | | |
|  | *enzymatic* | | |  | *Localisation in maize* | | |  | *Heterologous over-expression in Arabidopsis* | | | | |
| FNR isoforms | *K*_M_ Fd µM | *k*_cat_  s^-1^ | pI | | location, on cell rupture | Cyt_b_*f* co-purification | maize tether | | Interactions with Arabidopsis tether | | Impact on photosynthetic electron transport |  |  |
| ZmFNR1 | 2.7 | 174 | 6.4 | | membrane bound only | co-purified | TROL | | TROL | | increased ФI/ФII on dark to light transition, faster NPQ development | 2, 5 |  |
| ZmFNR2 | 3.4 | 288 | 5.4 | | membrane bound and soluble | co-purified | TROL | | Tic62 | | decreased ФI/ФII on dark to light transition, slower NPQ development | 2, 5 |  |
| ZmFNR3 | 2.7 | 261 | 5.4 | | soluble only | not-co-purified | none | | none | | decreased ФI/ФII on dark to light transition, slower NPQ development | 2, 5 |  |
