# Supplementary material for: Regulation of photosynthetic electron flow on dark to light transition by ferredoxin:NADP(H) oxidoreductase interactions
Source: eLife. 2021 Mar 9;10:e56088. doi: 10.7554/eLife.56088 (PMC7984839; doi:10.7554/eLife.56088)
Supplement: Supplementary file 2. — (a) Table of mixed effects model investigating changes in FNR density between different chloroplast sub-compartments in WT Arabidopsis. Analysis of data presented in Figure 1—figure supplement 2. Fixed effects taking either label density in the stroma as the intercept or label density in the margins/lamellae as the intercept. Linear mixed model fit by REML. Signif. codes: 0 ‘***’ 0.001 ‘**’ 0.01 ‘*’ 0.05 ‘.’ 0.1 ‘’ 1. (b) Table of mixed effects model investigating changes in cytochrome f density between different chloroplast sub-compartments in WT Arabidopsis. Analysis of data presented in Figure 1—figure supplement 2. Fixed effects taking either label density in the stroma as the intercept or label density in the margins/lamellae as the intercept. Linear mixed model fit by REML. Signif. codes: 0 ‘***’ 0.001 ‘**’ 0.01 ‘*’ 0.05 ‘.’ 0.1 ‘’ 1. (c) Table of fitting parameters and errors in comparison of light-dependent NADP+ reduction by different genotypes. Analysis performed using the data in Figure 4. Fits were calculated from experiments on individual chloroplast preparations, and then the parameters, and the fitting errors averaged. (d) Table of statistical analysis on the contribution of the fast phase to total amplitude of light-dependent fluorescence change in the chloroplast assay of NADP+ reduction. Analysis performed using the data averaged in Figure 4 and in (c). (e) Table of fitting parameters and errors in comparison of dark NADPH oxidation by different genotypes. Analysis performed using the data in Figure 4. Fits were calculated from experiments on individual chloroplast preparations, and then the parameters, and the fitting errors averaged. (f) Table of Pm values and statistical analysis of plants used for PAM analysis of the high light response. Analysis performed using the data in Figure 5, with example traces given in Figure 5—figure supplement 2. Pm determination of dark adapted leaves in order to calculate PSI parameters in response to high light t [file elife-56088-supp2.docx]

| **Supplementary File 2a. Table of Mixed Effects Model investigating changes in FNR density between different chloroplast sub-compartments in WT Arabidopsis.** Analysis of data presented in Figure 1 Supplement 2. Fixed Effects taking either label density in the stroma as the intercept or label density in the margins/lamellae as the intercept. Linear mixed model fit by REML. Signif. codes: 0 ‘***’ 0.001 ‘**’ 0.01 ‘*’ 0.05 ‘.’ 0.1 ‘ ’ 1 | | | | | | | | | | | | | | | | | | | |
| --- | --- | --- | --- | --- | --- | --- | --- | --- | --- | --- | --- | --- | --- | --- | --- | --- | --- | --- | --- |
| **Deletion test** carried out using using Satterthwaite’s method with the R package lmerTest (Kuznetsova, Brockhoff & Christensen 2017). The model is a mixed effects model with random intercepts. The square root of response is the response variable, tissue is the fixed effect and individual the random effect. | | | | | | | | | | | | | | | | | | | |
| Fixed effect deleted | Sum Sq | | Mean Sq | | | Num DF | | | Den DF | | | | F value | | | Pr (>F) | | |  |
| sub-compartment | 284.46 | | 94.82 | | | 3 | | | 66.825 | | | | 118.88 | | | 2.20^-16 | | | *** |
|  | | | | | | | | | | | | | | |  | |  | |  |
| **Model summary:** | | | | |  | | |  | | | |  | | |  | |  | |  |
| Random effects: | |  | | |  | | |  | | | |  | | |  | |  | |  |
| Groups | | Name | | | Variance | | | | | Std. Dev. | | | |  | |  |  | |  |
| individual | | (Intercept) | | | 0.01648 | | | | | 0.1284 | | | |  | |  |  | |  |
| Residual | |  | | | 0.79758 | | | | | 0.8931 | | | |  | |  |  | |  |
| Number of obs: 91, groups: individual, 25 | | | | | | | | | | | | | | |  | |  | |  |
| Fixed effects when stroma is set as the intercept: | | | | | | | | | | | | | | | | | | | |
|  | | Estimate | | Std. Error | | | DF | | | | t value | | | | Pr (>\|t\|) | | |  |  |
| (Intercept) | | 2.3925 | | 0.1924 | | | 86.9198 | | | | 12.438 | | | | <2.00^-16 | | | *** |  |
| cytosol | | -2.3791 | | 0.2612 | | | 67.8887 | | | | -9.107 | | | | 2.19^-13 | | | *** |  |
| grana | | -0.2515 | | 0.2693 | | | 65.7707 | | | | -0.934 | | | | 0.354 | | |  |  |
| margin/lamellae | | 2.5477 | | 0.2693 | | | 65.7707 | | | | 9.462 | | | | 6.86^-14 | | | *** |  |
|  | |  | | |  | | |  | | | |  | | |  | |  | |  |
| Fixed effects when margin/lamellae is set as the intercept: | | | | | | | | | | | | | | | | | | | |
|  | | Estimate | | Std. Error | | | DF | | | | | t value | | | Pr (>\|t\|) | | | |  |
| (Intercept) | | 4.9402 | | 0.1924 | | | 86.9198 | | | | | 25.683 | | | <2.00^-16 | | | *** | |
| cytosol | | -4.9269 | | 0.2612 | | | 67.8887 | | | | | -18.86 | | | <2.00^-16 | | | *** | |
| grana | | -2.7993 | | 0.2693 | | | 65.7707 | | | | | -10.396 | | | 1.62^-15 | | | *** | |
| stroma | | -2.5477 | | 0.2693 | | | 65.7707 | | | | | -9.462 | | | 6.86^-14 | | | *** | |

| **Supplementary File 2b. Mixed Effects Model investigating changes in cytochrome *f* density between different chloroplast sub-compartments in WT Arabidopsis.** Analysis of data presented in Figure 1 Supplement 2. Fixed Effects taking either label density in the stroma as the intercept or label density in the margins/lamellae as the intercept. Linear mixed model fit by REML. Signif. codes: 0 ‘***’ 0.001 ‘**’ 0.01 ‘*’ 0.05 ‘.’ 0.1 ‘ ’ 1 | | | | | | | | | | | | | | | | | | | |
| --- | --- | --- | --- | --- | --- | --- | --- | --- | --- | --- | --- | --- | --- | --- | --- | --- | --- | --- | --- |
| **Deletion test** carried out using using Satterthwaite’s method with the R package lmerTest (Kuznetsova, Brockhoff & Christensen 2017). The model is a mixed effects model with random intercepts. The square root of response is the response variable, tissue is the fixed effect and individual the random effect. | | | | | | | | | | | | | | | | | | | |
| Fixed effect deleted | Sum Sq | | Mean Sq | | | Num DF | | | Den DF | | | | F value | | | Pr (>F) | | |  |
| sub-compartment | 15.211 | | 5.0702 | | | 3 | | | 39 | | | | 23.586 | | | 7.135e-09 | | | *** |
|  | |  | | |  | | |  | | | |  | | |  | |  | |  |
| **Model summary**: | | | | | | | | | | | | | | | | | | | |
| Random effects: | |  | | |  | | |  | | | |  | | |  | |  | |  |
| Groups | | Name | | | Variance | | | | | Std. Dev. | | | |  | |  |  | |  |
| individual | | (Intercept) | | | 0.007045 | | | | | 0.08393 | | | |  | |  |  | |  |
| Residual | |  | | | 0.214969 | | | | | 0.46365 | | | |  | |  |  | |  |
| Number of obs: 56, groups: individual, 14 | | | | | | | | | | | | | | |  | |  | |  |
|  | |  | | |  | | |  | | | |  | | |  | |  | |  |
| Fixed effects when stroma is set as the intercept: | | | | | | | | | | | | | | | | | | | |
|  | | Estimate | | Std. Error | | | DF | | | | t value | | | | Pr (>\|t\|) | | |  |  |
| (Intercept) | | 0.283 | | 0.1259 | | | 51.8434 | | | | 2.247 | | | | 0.0289 | | | * |  |
| relevel cytosol | | -0.2765 | | 0.1752 | | | 39 | | | | -1.578 | | | | 0.1227 | | |  |  |
| relevel grana | | 0.863 | | 0.1752 | | | 39 | | | | 4.925 | | | | 1.59^-05 | | | *** |  |
| relevel margin/lamellae | | 0.9072 | | 0.1752 | | | 39 | | | | 5.177 | | | | 7.15^-06 | | | *** |  |
|  | |  | | |  | | |  | | | |  | | |  | |  | |  |
| Fixed effects when margin/lamellae is set as the intercept: | | | | | | | | | | | | | | | | | | | |
|  | | Estimate | | Std. Error | | | DF | | | | | t value | | | Pr (>\|t\|) | | | |  |
| (Intercept) | | 1.19021 | | 0.12593 | | | 51.84340 | | | | | 9.451 | | | 7.24^-13 | | | *** | |
| relevel cytosol | | 1.18371 | | 0.17524 | | | 39.00000 | | | | | -6.755 | | | 4.66^-08 | | | *** | |
| relevel grana | | -0.04422 | | 0.17524 | | | 39.00000 | | | | | -0.252 | | | 0.802 | | |  | |
| relevel stroma | | -0.90724 | | 0.17524 | | | 39.00000 | | | | | -5.177 | | | 7.15^-06 | | | *** | |

| **Supplementary File 2c. Fitting parameters and errors in comparison of light dependent NADP^+^ reduction by different genotypes.**  Analysis performed using the data in Figure 4. Fits were calculated from experiments on individual chloroplast preparations, and then the parameters, and the fitting errors averaged. | | | | | | | | | | | | |
| --- | --- | --- | --- | --- | --- | --- | --- | --- | --- | --- | --- | --- |
|  | Fitting parameters  Parameters calculated individually for 3-6 biological replicates and then averaged ± s.d. | | | | | | Errors (absolute, 95% confidence)  Errors in the fit calculated individually for 3-6 biological replicates and then averaged ± s.d. | | | | | |
|  | $F_{\infty}$ | $A_{fast}$ | $A_{slow}$ | $k_{fast}$ | $k_{slow}$ |  | | $\Delta F_{\infty}$ | $\Delta A_{fast}$ | $\Delta A_{slow}$ | $\Delta k_{fast}$ | $\Delta k_{slow}$ |
| wt | 0.0134  ±0.0072 | 0.697  ±0.048 | 0.303  ±0.048 | 3.74  ±1.62 | 0.111  ±0.052 |  | | 0.000677  ±0.000465 | 0.0674  ±0.0455 | 0.0272  ±0.0144 | 1.51  ±0.45 | 0.0758  ±0.0576 |
| *fnr1* | 0.0155  ±0.0075 | 0.865  ±0.117 | 0.135  ±0.117 | 10.8  ±6.62 | 0.235  ±0.252 |  | | 0.000185  ±6.48E-05 | 0.0371  ±0.0338 | 0.00949  ±0.00889 | 4.88  ±3.40 | 0.100  ±0.110 |
| *fnr1*-ZmFNR1 | 0.0201  ±0.0082 | 0.701  ±0.060 | 0.298  ±0.06 | 10.2  ±5.62 | 0.300  ±0.122 |  | | 0.000243  ±0.000101 | 0.0597  ±0.0390 | 0.0239  ±0.0196 | 3.46  ±1.70 | 0.0842  ±0.0384 |
| *fnr1-*ZmFNR2 | 0.0158  ±0.0037 | 0.696  ±0.084 | 0.304  ±0.085 | 8.60  ±3.12 | 0.388  ±0.225 |  | | 0.00021  ±2.5E-05 | 0.0705  ±0.0230 | 0.0367  ±0.0187 | 5.44  ±3.26 | 0.106  ±0.076 |
| *fnr1-*ZmFNR3 | 0.0164  ±0.0067 | 0.833  ±0.109 | 0.167  ±0.120 | 9.04  ±5.18 | 0.209  ±0.119 |  | | 0.000213  ±1.31E-05 | 0.0552  ±0.0120 | 0.0159  ±0.0049 | 4.34  ±3.34 | 0.106  ±0.053 |

| **Supplementary File 2d. Statistical analysis on the contribution of the fast phase to total amplitude of light dependent fluorescence change in the chloroplast assay of NADP^+^ reduction.** Analysis performed using the data in Figure 4 and Averaged in Supplementary File 2c.  One Way Analysis of Variance | | | | | | | | | | | | | |
| --- | --- | --- | --- | --- | --- | --- | --- | --- | --- | --- | --- | --- | --- |
| Normality Test (Shapiro-Wilk): Passed (P = 0.642) | | | | | | | | | | | | | |
| Equal Variance Test (Brown-Forsythe): Passed (P = 0.629) | | | | | | | | | | | | | |
|  | *n* | | *missing* | | | *mean* | | *Std Dev* | | | *SEM* | | |
| wt | 3 | | 0 | | | 0.697 | | 0.0479 | | | 0.0276 | | |
| *fnr1* | 3 | | 0 | | | 0.865 | | 0.117 | | | 0.0675 | | |
| *fnr1*-ZmFNR1 | 4 | | 0 | | | 0.702 | | 0.0600 | | | 0.0300 | | |
| *fnr1-*ZmFNR2 | 5 | | 0 | | | 0.696 | | 0.0848 | | | 0.0379 | | |
| *fnr1-*ZmFNR3 | 5 | | 0 | | | 0.833 | | 0.110 | | | 0.0490 | | |
|  | | | | | | | | | | | | | |
| Source of Variation | | *DF* | | | *SS* | | *MS* | | *F* | | | *P* | |
| Between Groups | | 5 | | | 0.114 | | 0.0228 | | 2.995 | | | 0.043 | |
| Residual | | 16 | | | 0.122 | | 0.00761 | |  | |  | | |
| Total | | 21 | | | 0.236 | |  | |  | |  | | |
|  | | | | | | | | | | | | | |
| The differences in the mean values among the treatment groups are greater than would be expected by chance; there is a statistically significant difference (P = 0.043). | | | | | | | | | | | | | |
| Power of performed test with alpha = 0.050: 0.514 | | | | | | | | | | | | | |
|  | | | | | | | | | | | | | |
| Multiple Comparisons versus Control Group (Holm-Sidak method): | | | | | | | | | | | | | |
|  | | | | | | | | | | | | | |
| Comparison | | | | Diff of Means | | | | | | t | | | P |
| wt vs. *fnr1* | | | | 0.169 | | | | | | 2.368 | | | 0.145 |
| wt vs. *fnr1-*ZmFNR3 | | | | 0.136 | | | | | | 2.136 | | | 0.180 |
| wt vs. *fnr1*-ZmFNR1 | | | | 0.00503 | | | | | | 0.0756 | | | 0.996 |
| wt vs. *fnr1*-ZmFNR2 | | | | 0.000679 | | | | | | 0.0107 | | | 0.992 |

| **Supplementary File 2e. Fitting parameters and errors in comparison of dark NADPH oxidation by different genotypes.**  Analysis performed using the data in Figure 4. Fits were calculated from experiments on individual chloroplast preparations, and then the parameters, and the fitting errors averaged. | | | | | | | |
| --- | --- | --- | --- | --- | --- | --- | --- |
|  | Fitting parameters (Hill)  Parameters calculated individually for 3-6 biological replicates and then averaged ± s.d. | |  | Errors (absolute, 95% confidence)  Errors in the fit calculated individually for 3-6 biological replicates and then averaged ± s.d. | | | |
|  | $F_{\infty}'$ | $k_{rec}$ | $n$ |  | $\Delta F_{\infty}'$ | $\Delta k_{rec}$ | $\Delta n$ |
| wt | -0.00344 ±0.00112 | 0.415±0.0980 | 1 |  | 0.000619  ±7.2E-05 | 0.109  ±0.038 | - |
| *fnr1* | -0.00341±0.00414 | 0.455±0.410 | 1 |  | 0.000694  ±0.00013 | 0.141  ±0.1812 | - |
| *fnr1-* ZmFNR1 | -6.38E-04±0.00147 | 0.202±0.098 | 1 |  | 0.00103  ±0.00052 | 0.0406  ±0.0115 | - |
| *fnr1-* ZmFNR2 | -0.00361±0.00196 | 0.373±0.034 | 1 |  | 0.00070  ±0.00013 | 0.0966  ±0.0145 | - |
| *fnr1-* ZmFNR3 | -0.00287±0.0029 | 0.185±0.034 | 1 |  | 0.00089  ±0.00011 | 0.0312  ±0.0005 | - |

| **Supplementary File 2f. Pm values and statistical analysis of plants used for PAM analysis of the high light response.** Analysis performed using the data in Figure 5, with example traces given in Figure 5 Supplement 1. Pm determination of dark-adapted leaves in order to calculate PSI parameters in response to high light treatment of Wt, *fnr1*, and *fnr1* plants expressing either ZmFNR1, ZmFNR2 or ZmFNR3 Arabidopsis plants (see Figure 5). n = 5-7 replicates. Signif. codes: 0 ‘***’ 0.001 ‘**’ 0.01 ‘*’ 0.05 ‘.’ 0.1 ‘ ’ 1 | | | | | | | |
| --- | --- | --- | --- | --- | --- | --- | --- |
|  | | | Mean ± sd | | | | |
| wt | | | 1.04 | ±0.07 | |  |  |
| *fnr1* | | | 0.91 | ±0.25 | |  |  |
| *fnr1-* ZmFNR1 | | | 1.11 | ±0.22 | |  |  |
| *fnr1-* ZmFNR2 | | | 1.05 | ±0.23 | |  |  |
| *fnr1-* ZmFNR3 | | | 1.11 | ±0.23 | |  |  |
|  | | |  |  | |  |  |
| ANOVA (One - way ) and Post hoc TSD Tukey test of the Pm determination for assessing the PSI responses to high light treatment on leaves from wild type, *fnr1*, and *fnr1* plants expressing either ZmFNR1, ZmFNR2 or ZmFNR3 (n>6 replicates). Signif. codes: 0 ‘***’ 0.001 ‘**’ 0.01 ‘*’ 0.05 ‘.’ 0.1 ‘ ’ 1 in .  Overall ANOVA | | | | | | | |
|  | Df | | Sum Sq | | Mean Sq | F value | Pr(>F) |
| genotype | 4 | | 0.3142 | | 0.07854 | 1.719 | 0.16 |
| Residuals | 52 | | 2.3757 | | 0.04569 |  |  |
| Post hoc TSD Tukey | | | | |  |  |  |
|  | | Estimate | | | Std. Error | t value | Pr(>\|t\|) |
| ZmFNR1-fnr1 | | 0.193861 | | | 0.094252 | 2.057 | 0.254 |
| ZmFNR2-*fnr1* | | 0.135583 | | | 0.08726 | 1.554 | 0.533 |
| ZmFNR3-*fnr1* | | 0.200417 | | | 0.085565 | 2.342 | 0.148 |
| wt-*fnr1* | | 0.096689 | | | 0.089221 | 1.084 | 0.814 |
| ZmFNR2-ZmFNR1 | | -0.058278 | | | 0.094252 | -0.618 | 0.971 |
| ZmFNR3-ZmFNR1 | | 0.006556 | | | 0.092685 | 0.071 | 1 |
| wt-ZmFNR1 | | -0.097172 | | | 0.09607 | -1.011 | 0.849 |
| ZmFNR3-ZmFNR2 | | 0.064833 | | | 0.085565 | 0.758 | 0.941 |
| wt-ZmFNR2 | | -0.038894 | | | 0.089221 | -0.436 | 0.992 |
| wt-ZmFNR3 | | -0.103727 | | | 0.087565 | -1.185 | 0.76 |
