# Supplementary material for: Regulation of photosynthetic electron flow on dark to light transition by ferredoxin:NADP(H) oxidoreductase interactions
Source: eLife. 2021 Mar 9;10:e56088. doi: 10.7554/eLife.56088 (PMC7984839; doi:10.7554/eLife.56088)
Supplement: Transparent reporting form [file elife-56088-transrepform.pdf]

eLIFE
1st Floor
24 Hills Road
Cambridge CB2 1JP, UK
P 01223 855340
W elifesciences.org
T @elife

eLife's transparent reporting form

We encourage authors to provide detailed information *within their submission* to facilitate the interpretation and replication of experiments. Authors can upload supporting documentation to indicate the use of appropriate reporting guidelines for health-related research (see EQUATOR Network), life science research (see the BioSharing Information Resource), or the ARRIVE guidelines for reporting work involving animal research. Where applicable, authors should refer to any relevant reporting standards documents in this form.

If you have any questions, please consult our Journal Policies and/or contact us: editorial@elifesciences.org.

Sample-size estimation

- You should state whether an appropriate sample size was computed when the study was being designed
- You should state the statistical method of sample size computation and any required assumptions
- If no explicit power analysis was used, you should describe how you decided what sample (replicate) size (number) to use

Please outline where this information can be found within the submission (e.g., sections or figure legends), or explain why this information doesn't apply to your submission:

For immunogold labelling: The minimum number of chloroplasts necessary for a statistically sound interpretation was identified using a Power Analysis of a preliminary data set (25 chloroplasts) in a Mixed Model test (chosen as a statistical test because the protein density in different sub-compartments of each chloroplast is related). This was defined as 3 chloroplasts per individual, but we have analysed 15 to generate as much statistical power as possible. This is described in both the methods section and the results text.

No power analysis was performed for other methods, but we judged that if results were consistent between 3 separate repetitions of western blotting with 3 separate groups of individuals, and between 3 separate experiments on NADP(H) kinetics with chloroplasts made from 3 separate groups of individuals the results were worth consideration. The same is true for measurements on individual plants, where we judged that averages of 6-12 individuals were sufficient, if they were consistent between 2-3 experiments performed on separate occasions

Replicates

- You should report how often each experiment was performed
- You should include a definition of biological versus technical replication
- The data obtained should be provided and sufficient information should be provided to indicate the number of independent biological and/or technical replicates
- If you encountered any outliers, you should describe how these were handled
- Criteria for exclusion/inclusion of data should be clearly stated

eLife Sciences Publications, Ltd is a limited liability non-profit non-stock corporation incorporated in the State of Delaware, USA, with company number 5030732, and is registered in the UK with company number FC030576 and branch number BR015634 at the address 1st Floor, 24 Hills Road, Cambridge CB2 1JP | August 2014

- High-throughput sequence data should be uploaded before submission, with a private link for reviewers provided (these are available from both GEO and ArrayExpress)

Please outline where this information can be found within the submission (e.g., sections or figure legends), or explain why this information doesn't apply to your submission:

For Immunogold labelling, in analysis of the first data set and pilot study (Supplementary Figure 1), and chloroplast area (Supplementary Figure 3) we have plotted the values for individual chloroplasts. Outliers are shown as dots on the graph. For comparisons of area including the stromal lamellae as a single measurement (Figures 3 and 7), the area of this region was frequently so small that no density was detected. This skewed the data heavily with many zeros, and so we combined the total area and protein detection in 15-22 chloroplasts for each of three biological replicates (individual plants), and present statistical analysis and the average of these three genotypes. Again, outliers are shown as dots.

For Western blotting: Experiments were performed at least 3 times, using samples from different individuals, or in the case of experiments on chloroplasts, on new mixtures of individuals of the same genotype each time.

For measurements of NADP(H) kinetics, the experiments were performed at least 3 times for each genotype, using at least 5 individual plants for each single chloroplast preparation. Traces were manually curated to remove the first and last measurements if necessary, as these were occasionally anomalous. Traces are averages of 15-20 technical replicate measurements on a single chloroplast preparation. Data fitting is on such an average trace from a representative chloroplast preparation for each individual.

Electrochromic bandshift measurements are averages of 5-7 individuals, and the experiment was performed on two separate occasions with similar results. Chlorophyll fluorescence measurements were are averages of 6-12 individuals, and the experiment was repeated on 3 separate occasions, with similar results. Typical original traces are shown in Supplementary Figure 6, so that readers can judge the accuracy of the measurements.

eLife Sciences Publications, Ltd is a limited liability non-profit non-stock corporation incorporated in the State of Delaware, USA, with company number 5030732, and is registered in the UK with company number FC030576 and branch number BR015634 at the address 1st Floor, 24 Hills Road, Cambridge CB2 1JP | August 2014

Statistical reporting

- Statistical analysis methods should be described and justified
- Raw data should be presented in figures whenever informative to do so (typically when N per group is less than 10)
- For each experiment, you should identify the statistical tests used, exact values of N, definitions of center, methods of multiple test correction, and dispersion and precision measures (e.g., mean, median, SD, SEM, confidence intervals; and, for the major substantive results, a measure of effect size (e.g., Pearson's r, Cohen's d)
- Report exact p-values wherever possible alongside the summary statistics and 95% confidence intervals. These should be reported for all key questions and not only when the p-value is less than 0.05.

Please outline where this information can be found within the submission (e.g., sections or figure legends), or explain why this information doesn't apply to your submission:

Statistical analysis using a mixed effects model is described in the materials and methods section — briefly, the presence of multiple measures for each individual means that there is non-independence present in the data, which was corrected for by using a mixed effects model with individual as a random effect. Individual contrasts were interpreted using the treatment contrasts presented in the summary table given by the R lme4 and lmerTest packages for each model. For this analysis, statistical tables are provided with p values for all comparisons.

For chlorophyll fluorescence and electrochromic bandshift, all values found to be significant in a post-hoc analysis of variance (Anova) are indicated on the graphs. P-values are indicated with star numbers (details in the legend).

(For large datasets, or papers with a very large number of statistical tests, you may upload a single table file with tests, Ns, etc., with reference to sections in the manuscript.)

Group allocation

- Indicate how samples were allocated into experimental groups (in the case of clinical studies, please specify allocation to treatment method); if randomization was used, please also state if restricted randomization was applied
- Indicate if masking was used during group allocation, data collection and/or data analysis

Please outline where this information can be found within the submission (e.g., sections or figure legends), or explain why this information doesn't apply to your submission:

Samples were grouped according to genotype (details in legends and figures). Randomization was not used as all plants were grown in identical conditions.

Additional data files ("source data")

- We encourage you to upload relevant additional data files, such as numerical data that are represented as a graph in a figure, or as a summary table
- Where provided, these should be in the most useful format, and they can be uploaded as "Source data" files linked to a main figure or table
- Include model definition files including the full list of parameters used
- Include code used for data analysis (e.g., R, MatLab)
- Avoid stating that data files are "available upon request"

eLife Sciences Publications, Ltd is a limited liability non-profit non-stock corporation incorporated in the State of Delaware, USA, with company number 5030732, and is registered in the UK with company number FC030576 and branch number BR015634 at the address 1st Floor, 24 Hills Road, Cambridge CB2 1JP | August 2014

Please indicate the figures or tables for which source data files have been provided:

The fitting model for NADP(H) kinetics and all parameters used are explained in the methods section.
